# Implementation and validation of an in‐house geometry optimization software for SRS VMAT planning of multiple cranial metastases

**DOI:** 10.1002/acm2.12961

**Published:** 2020-07-06

**Authors:** LiCheng Kuo, PengPeng Zhang, Hai Pham, Åse M. Ballangrud

**Affiliations:** ^1^ Department of Medical Physics Memorial Sloan Kettering Cancer Center New York NY USA

**Keywords:** cranial, geometry optimization, multiple metastases, SRS, VMAT

## Abstract

**Purpose:**

The implementation and evaluation of an in‐house developed geometry optimization (GO) software are described. The GO script provides optimal lesion clustering, isocenter placement, and collimator angle of each arc for cranial multi‐lesion stereotactic radiosurgery (SRS) volumetric modulated arc therapy (VMAT) planning.

**Materials and methods:**

An Eclipse‐plugin program was developed to facilitate automatic plan geometry generation for multiple metastases SRS VMAT plans. A mixed, semi‐supervised exhaustive and k‐means clustering method is used to group lesions and place isocenters. The sum of squared euclidean distance (SSED) and the boundaries of lesions’ projection from beams’ eye view are used as supervised parameters to determine the optimal isocenter numbers. The collimator angle is optimized by minimizing the sum of the MLC opening area from all gantry angles for each arc. In all, 10 clinical cases treated during 2016–2017 were compared to plan quality of GO script generated plans. Paddick gradient index (GI), conformity index (CI), and local brain volume receiving 12 Gy (local V12 Gy) around each lesion were compared.

**Result:**

For four cases, the number of isocenters was reduced in the GO plans. For four other cases, the GO plans had the same number of isocenters as their corresponding clinical plans but with different lesion grouping. The GO plans had significantly lower GI (4.1 ± 1.0 vs 4.4 ± 0.9, *P* < 0.0001) and local V12 Gy (5.1 ± 4.2 vs 5.5 ± 4.3 in cm^3^, *P* < 0.0001), but not significantly different mean normal brain dose or CI. The volume of normal brain receiving ≥6 Gy was significantly lower in the GO plans. The total time to run the GO script for each case was <2 min.

**Conclusion:**

The GO software automates lesion grouping, isocenter placement, and the collimator angles for SRS VMAT planning. When tested on 10 cases, the GO script resulted in improved plan quality and shorter planning time when compared to the clinical SRS VMAT plans.

## 1 INTRODUCTION

Radiation treatment of multiple metastatic cranial lesions with volumetric modulated arc therapy (VMAT) has become one of many radiation treatment options in the past years.^1^, ^2^, ^3^, ^4^, ^5^ Compared to conventional techniques such as Gamma Knife (Elekta, Crawley, UK), CyberKnife (Accuray Inc, Sunnyvale, CA, USA), and conventional C‐arm linear accelerators (Linacs) employing cones or conformally shaped multi‐leaf collimator (MLC) patterns, all targeting one lesion at a time; VMAT techniques improve the treatment delivery efficiency using one isocenter to target multiple lesions. These VMAT plans can achieve highly conformal dose distributions similar to Gamma Knife plans.^4^, ^5^, ^6^, ^7^ Although most of these studies were based on using a single isocenter VMAT plan to treat multiple cranial lesions,^1^, ^2^, ^3^, ^4^, ^5^, ^6^, ^7^, ^8^ other studies indicate that VMAT plans with multiple isocenters may be required to improve the plan quality, reduce the risk of comprised coverage for lesions far from isocenter,^9^, ^10^, ^11^ and account for the MLC model inaccuracy in the dose calculation algorithms.^12^, ^13^ Grouping the targets into multiple plans and isocenters results in increased treatment planning complexity and long planning times. It is challenging and time‐consuming for planners to determine the best grouping of lesions into separate isocenters, to select the best arcs for each isocenter, and finally choose the optimal collimator angles for each arc. Each planner may choose different solutions for the same patient, resulting in plans with variable plan quality and posing a challenge for institutional quality assurance.

Previous studies propose different methods to solve lesion grouping and optimal collimator geometry separately.^8^, ^14^, ^15^ However, none of them have presented the results from combining both methods. In this study, we present a tool for optimizing lesion grouping and finding the optimal collimator angle for each arc so that SRS VMAT treatment plans are generated in shorter time and with more consistent plan quality. We prototyped a geometry optimization (GO) script in Matlab (Mathworks, Natick, MA, USA), subsequently implemented it using the Eclipse Scripting Application Programming interface (ESAPI, Varian Medical Systems, Palo Alto, CA), and eventually integrated it into the production Eclipse system for routine clinical operations. The overall goal of this script is to reduce planning time while creating plan geometries that can produce plans that at least match or improve on manually created plans. In this study, we present a thorough description of this script and the script‐generated plans are validated against a set of high‐quality, manually created clinical plans.

## 2 MATERIALS AND METHODS

### 2.A Automatic lesion grouping algorithm

**Fig. 1:**
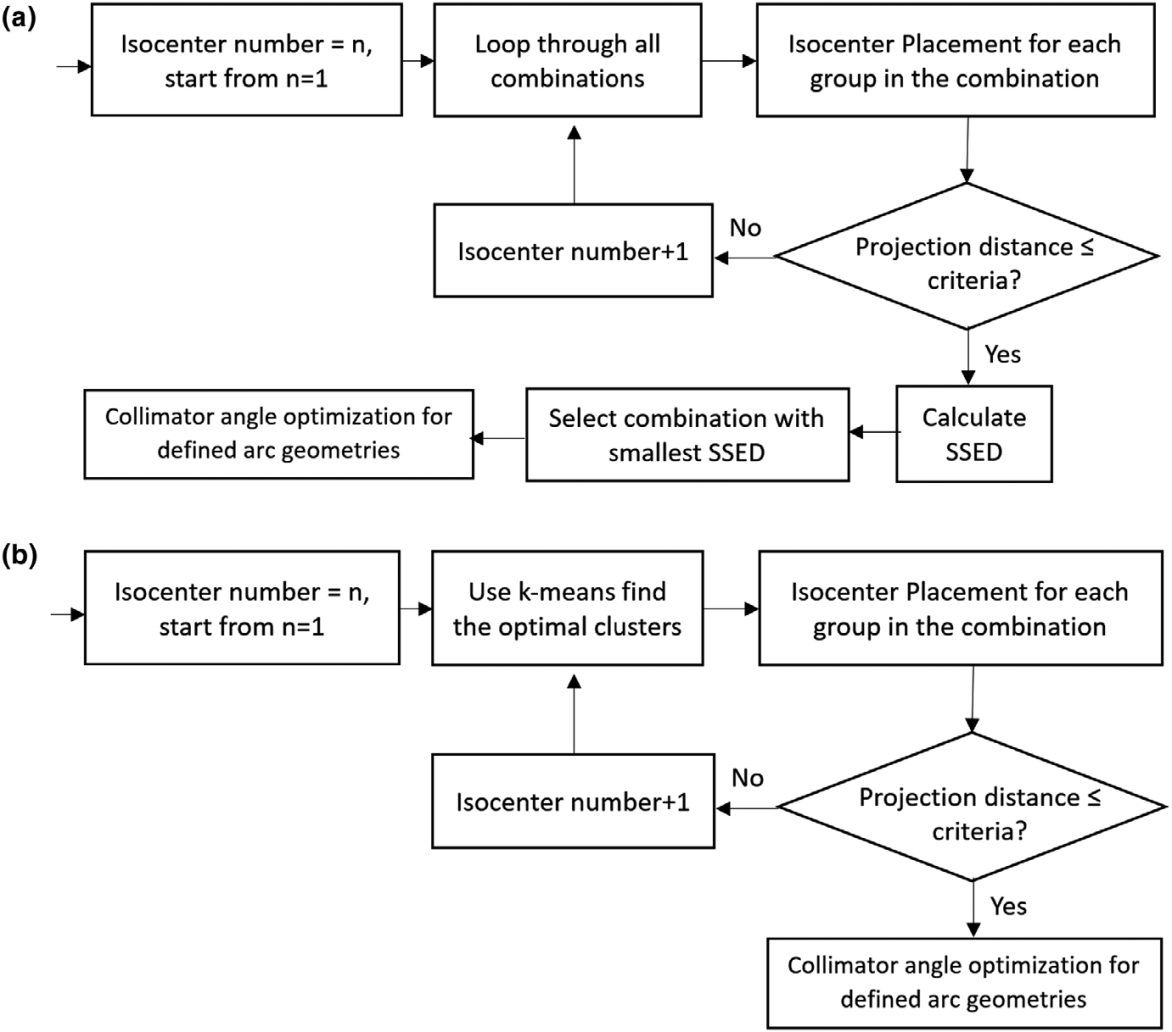
Illustration of (a) the exhaustive search and (b) k‐means methods for isocenter number selection. Minimizing within‐cluster variance (equivalent to SSED) is included in k‐means++ algorithm.

In this study, we developed a mixed, semi‐supervised exhaustive and k‐means clustering method to group lesions and place isocenters. For total lesion numbers less or equal to 7 and isocenter numbers less or equal to 3, the script uses the exhaustive search method, and for more than 7 lesions the k‐means method is used. We choose to combine both methods in this study since the exhaustive method is not very efficient compared to the k‐means method when the number of lesions is large. Both methods use the squared Euclidean distance as the optimization metric, theoretically they should give the same clustering results. The flowchart for both methods is shown in Fig. 1.

For the exhaustive search method, the search starts with one isocenter (one cluster), and lesions are grouped based on exhaustive combination lists predefined for up to n = 3 isocenters, which for a single isocenter is the trivial single combination of all lesions being in the same cluster. In every possible combination, an isocenter location is placed for each of n clusters based on the lesions’ centroid in that cluster. A projection distance check is performed to determine if all lesions fall within a preset distance criteria from the assigned isocenter. If none of the possible combinations meet the criteria, the number of isocenters is increased by one, and the process is repeated, with all the possible combinations of grouping lesions into n + 1 clusters with n + 1 isocenters being tested again, until one or more combinations are found where all clusters meet the geometric distance criteria. If multiple different combinations of clusters meet the criteria, the Sum of squared euclidean distance (SSED) from the isocenter to all grouped lesions is calculated for each group and the combination with the smallest SSED is selected as the optimal clustering result. Details of the isocenter placement and geometric distance check method will be described in the following sections.

If the total number of lesions is larger than 7 and the resulting number of isocenters is large than 3, a k‐means ++ clustering algorithm is used to group the lesions instead of the exhaustive method, running repeats equal to the total number of lesions to avoid suboptimal clustering results. We select squared Euclidean distance as the distance metric in the k‐means ++ algorithm to minimize the within‐cluster variance which is equivalent to the SSED. Each search starts with one isocenter per cluster and checks if each cluster generated from the k‐means method meets the distance criteria. If none of the clusters meet the criteria, the number of isocenters is increased by one as input for the next k‐means clustering until all clusters meet the set distance criteria.

### 2.B Isocenter placement

There are different methods to place isocenter for multiple lesions and a previous study compared the plan quality difference between these methods.^11^ These methods include volume centroid, centroid of equally weighted points, centroid of points weighted by inverse of volume, and treatment planning system built‐in method. In terms of dose fall‐off outside the target, there is no significant difference between these methods except for the inverse volume centroid method which was found to result in slightly inferior plan quality.^11^ The optimizer described in this study is placing the isocenter at the geometric center of the group. The isocenter is initially placed at the centroid of equally weighted points of the grouped lesions. Then the program adjusts the coordinates based on projected outer boundary (POB) of grouped lesions from the beam’s eye view (BEV) at gantry angles 0° and 270° with couch and collimator angle at 0° to make maximum boundary positions symmetric in superior/inferior, left and right, and anterior/posterior direction.

### 2.C Projection distance check

**Fig. 2:**
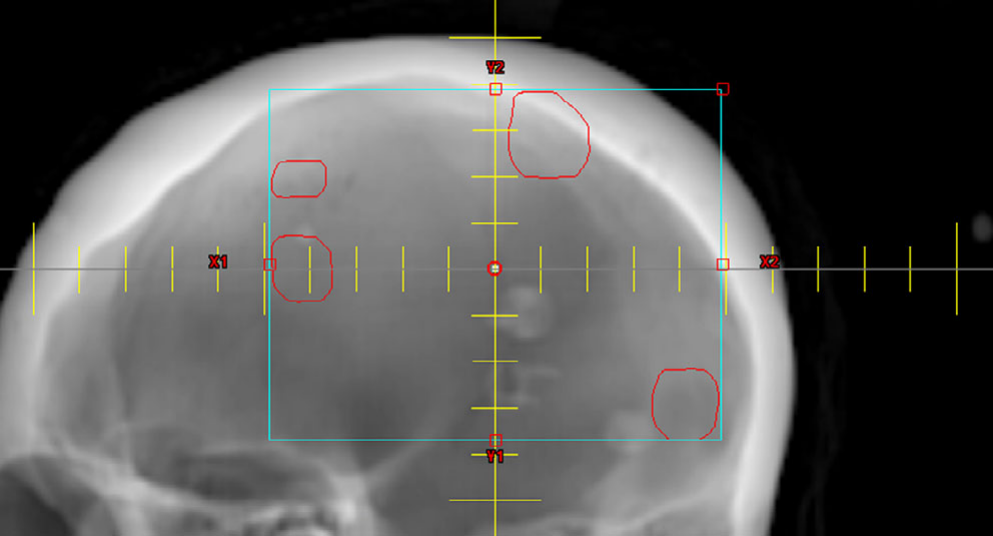
Illustration of the projection distance check. The red contours are the projected outer boundary of the grouped lesions in the beam's eye view (BEV). The maximum boundary distances from the isocenter in four directions are measured as D_boundary_ which must be smaller than D_max. boundary_. This figure is the 45° gantry BEV.

Instead of checking the distance from isocenter to the centroid of each lesion, this algorithm checks POB from the BEV of that isocenter at four different gantry angles: 0°, 45°, 270°, and 315° with collimator and couch angle at 0° (Fig. 2). The chosen default boundary distance criteria from isocenter for the Varian TrueBeam STx with HD120 MLC are <5 cm in each X direction and 4 cm in each Y direction, limiting the field to only use the 2.5 mm width MLC.^12^ For the Varian TrueBeam with Millennium 120 (M120) MLC, the chosen default distance criteria are less than 5 cm in both the X and Y directions. The 5 cm criteria is based on the recommendation from Morrison's,^9^ Roper's^10^ and Stanhope's^11^ studies. By checking the boundary distance from the BEV, we can maximize the utilization of MLC and minimize the trajectory of lesion projections beyond the distance criteria. The distance criteria in each of the X and Y directions can be changed from the default values in the settings prior to running the optimizer. In summary, the default maximum allowable distance from the BEV projection of grouped lesions to the isocenter, D_Max.boundary_, is

(1) $$D_{\text{Max}.\;\text{boundary}}\left\{\begin{array}{ccc} X1,X2: & \leq 5.0\;\text{cm} & \text{for HiDef and M}120\;\text{MLC}\\ Y1,Y2: & \leq 4.0\;\text{cm} & \text{for HiDef MLC}\\ Y1,Y2: & \leq 5.0\;\text{cm} & \text{for M}120\;\text{MLC} \end{array}\right\}$$

### 2.D Collimator angle optimization

**Fig. 3:**
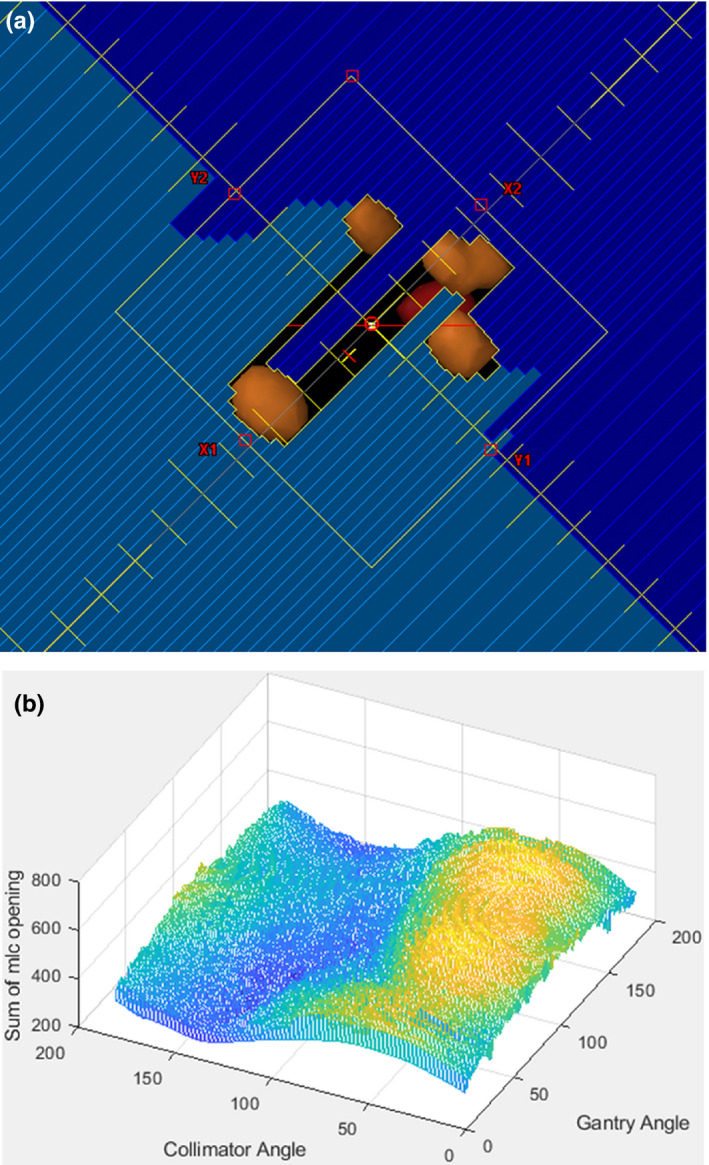
Collimator angle optimization method. (a) Multi‐leaf collimator (MLC) opening area which fit the grouped five lesions (orange) was calculated. (b) Sum of MLC opening area with different combinations of collimator and gantry angle for an example with five lesions.

Wu et al. calculated the total unblocked area by the MLC between lesions to optimize couch and collimator angles.^8^ In our study, the total MLC opening area defined by the POB in the BEV for each gantry angle of each manually selected arc is calculated with different collimator angles. The search range for collimator angles is from 0° to 165°, given by the machine limitations. Collimator angles from 195° to 0° are not included because the MLC leaves are symmetric. In this study, gantry and collimator angles calculation intervals are adjustable, with default set as 4° and 1°, respectively. In the final clinical implementation, however, the default is set to perform calculations at each control point for the gantry (approximately every 2°) and every 5° for the collimator. The total MLC opening area is summed along the entire range of gantry angles for each collimator angle. The optimal collimator angle has the smallest summed total MLC opening area (Fig. 3). The final jaw positions are fit to clustered lesions with optimal collimator angle for each manually selected arc using the method provided in the ESAPI. Maximum jaw positions in each Y direction are set to 4 cm for the Varian TrueBeam STx with HD120 to ensure that only the 2.5 mm MLC are used.

### 2.E Implementation in Varian Eclipse API 15.5

The software was originally developed in Matlab and rewritten in C# (Visual Studio 2017) as a Varian Eclipse plugin. The software development follows the IEEE guide to software requirements specifications (Std 830‐1993). One advancement of this program is the utilization of the write‐back feature of the Eclipse API v15.5. Plans with optimal isocenter setup and arc geometry created by the algorithm are directly written into the Eclipse database, eliminating the manual DICOM import step. Once created, the plans are ready in Eclipse for the planner to run dose optimization.

On startup, the GO script imports the current plan and structure set from Eclipse. The GO script will automatically select PTVs based on the PTV naming convention which includes the prescribed dose, and from this information the script predicts the fractionation for each PTV and groups them accordingly. The user can also manually select PTVs. The default machine for the new plan is the same as the current plan. The user can overwrite the default option by selecting a new machine. Once the isocenter optimization is completed, the GO script displays the calculated isocenter coordinates, lesions assigned to each isocenter, and the default arc geometry. The user can add, delete, or modify the default arc geometry. The GO script writes the optimized plans back into Eclipse if the user accepts the result. The GO script can also be used to optimize collimator angles for an existing plan in Eclipse. Once this option is selected, the GO script loads the arc geometries and beam information from the current plan in Eclipse. The information on the PTVs targeted in this plan is also loaded. Once the GO script completes the collimator optimization, the script displays the optimized collimator angle for each arc and writes the new collimator angles back to the existing plan in Eclipse.

### 2.F Pre‐clinical validation and release

Before releasing the plugin clinically, two tests were performed to validate that the GO script worked correctly and as intended. The first test confirmed that the algorithms were transcribed accurately by checking the consistency of isocenter and collimator optimization results between the Matlab code and the Eclipse API code. The second test was to check that all functions provided in the plugin worked properly. This included the import of patient data from Eclipse; automatic selection of PTVs before optimization; the ability of the isocenter optimizer to correctly group lesions and display grouped information based on the set geometric criteria; the ability of the collimator optimizer to optimize the angle based on default or user‐defined arc geometries for the grouped lesions; and the ability of the GO plugin script to write back plans with correct isocenter coordinates, machine, technique, MLC type, arc geometries, dose calculation algorithm, and jaw settings. The testing results were fully documented in the software development verification (SDV) document for independent review by the head of treatment planning and computer service. After the plugin was approved, it was released on a Saturday in February 2019, to avoid interference with clinical operation. The signed release form, as well as the project plan and SDV, were uploaded to a teamshare website for department‐wide references.

### 2.G Patient selection, treatment planning and plan quality comparison

In all, 10 clinical SRS VMAT cases with a total of 65 lesions treated from 2016 to 2017, each treated with three or more isocenters, were selected for testing of the GO software. Both the clinical and the GO plans were generated in Varian Eclipse V13.6 with a specific analytical anisotropic algorithm (AAA) dose calculation model tuned for small targets.^12^ The dose calculation grid was 1.25 mm. For cases where the GO software created identical lesion grouping and isocenter location to the actual clinical plan, the same couch angles and gantry angles used in the clinical plan were used for the GO plan but the GO plan used the optimal collimator angles as determined by the GO software. If the GO software created different lesion grouping than the clinical plan, four default arcs with couch angles at 0° (full arc), 90° ,45°, and 315° (180° range partial arcs) with the optimal collimator angles as determined by the GO software were used to generate the GO plan.

The plan quality of the GO plans was compared to the clinical plans by evaluating the Paddick gradient index (GI),^16^ RTOG GI,^17^ conformity index (CI),^18^ local brain volume receiving 12 Gy (local V12Gy) around each lesion, normal brain mean dose, and volumes of normal brain receiving 4 Gy–16 Gy in 1 Gy increments (V4Gy, V5Gy…V16Gy). In addition, total MU and calculation time for isocenter and collimator optimization were recorded for each case. A paired *T*‐test was used for statistical analysis.

## 3 RESULTS

The isocenter and collimator optimization algorithms were successfully translated into the GO script and gave the same results as the original Matlab code on grouping lesions and isocenter placement. Because the MLC fitting method in the Matlab code does not take the MLC travel motion limitation into account, for some arcs, the optimal collimator angle found by the GO script could be up to 15° different than in Matlab, but the final plan quality was almost identical (data are not shown).

For 4 out of the total 10 cases, the number of isocenters was reduced in the GO plans as compared to the clinical plans. For these cases, the total MUs were also reduced. For four other cases, the GO plans had the same number of isocenters as the clinical plans but with different lesion grouping (Table 1). For each of the 10 cases, the total geometry optimization time was <2 min. The time it takes for a planner to manually group the targets and select isocenter depends on the distribution and the number of lesions, and ranges from 20 to 60 min. The GO script will significantly shorten the treatment planning time for multi‐lesion SRS VMAT cases.^13^

**Table 1:** Numbers of isocenters and total MU for the 10 clinical plans and the corresponding GO plans.

	Number of lesions	Clinical plan number of isocenters	GO plan number of isocenters	Clinical plan total MUs	GO plan total MUs	GO optimization time (s)
Case 1	4	3	2	26 096	10 657	44.6
Case 2	7	3	3	15 069	9852	120.3
Case 3	5	3	3^a^	16 944	17 134	70.1
Case 4	7	3	3	15 565	18 272	110.8
Case 5	8	3	3^a^	17 787	17 836	66.9
Case 6	8	4	2	17 238	15 533	54.5
Case 7	7	3	3^a^	19 261	17 649	106.3
Case 8	5	3	2	16 477	10 461	96.1
Case 9	7	3	2	25 301	12 760	61.0
Case 10	7	3	3^a^	19 099	14 873	55.3

^a^ GO plan has different lesion grouping compared to the clinical plan.

**Fig. 4:**
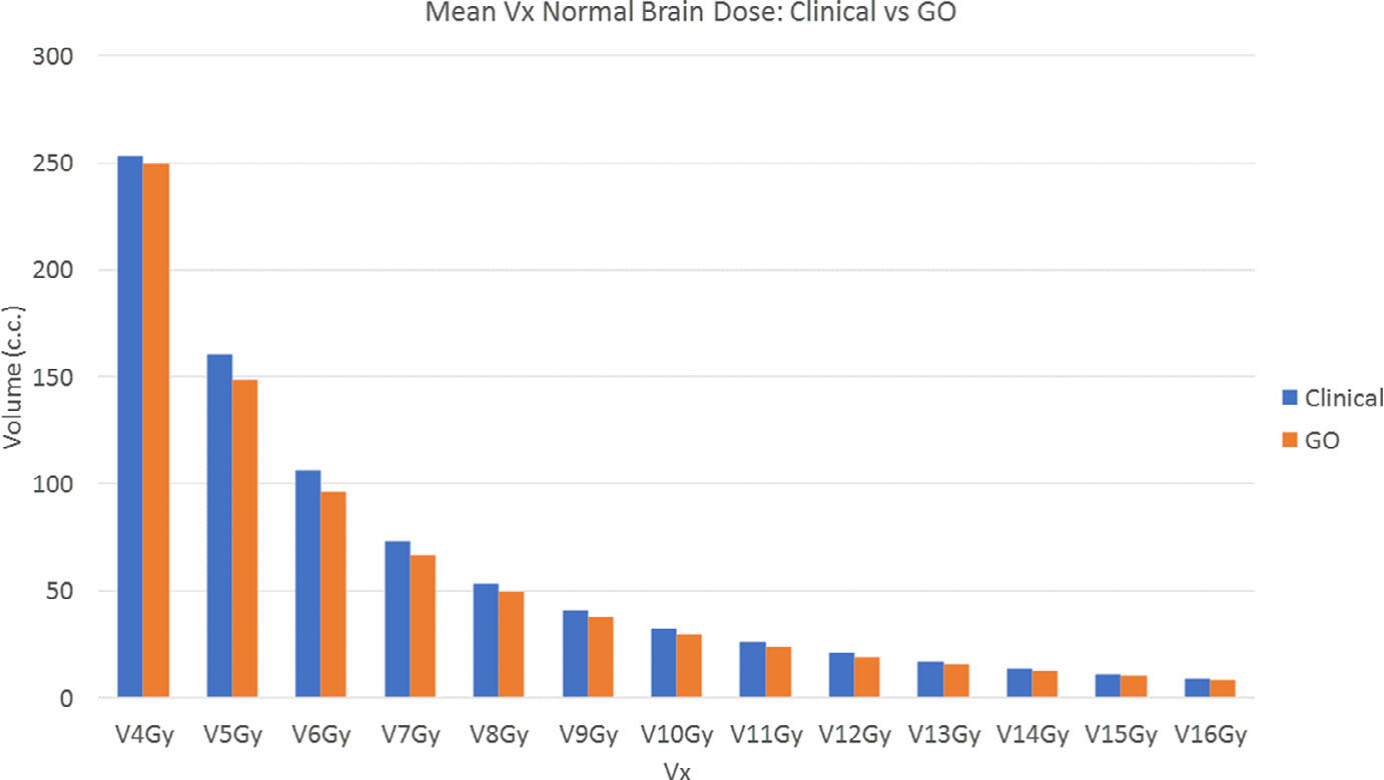
Comparison of mean Vx (mean volume receiving dose Gy) of normal brain between clinical plans and GO plans for 10 patients.

**Fig. 5:**
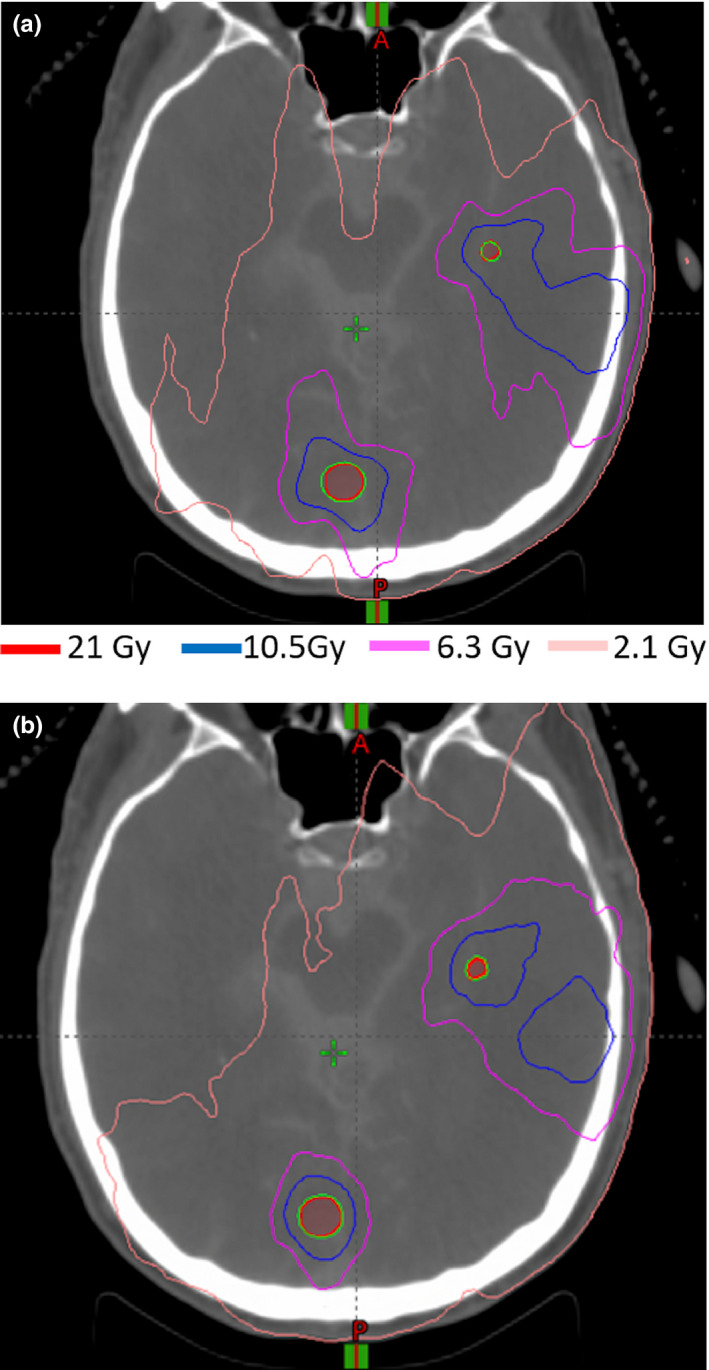
Isodose line comparison between the clinical plan (A) and the GO plan (B).

The Paddick‐GI, the RTOG‐GI, the CI, and the volume included in the local 12 Gy isodose line around each PTV are shown in Table 2, along with the dose to normal brain. The mean and range are listed for each parameter. A paired *T*‐test is used to compare the clinical plan to the GO plan for each parameter and the *P* value is shown in the last column. The GO plans had significantly lower GI, CI, and local V12Gy values than the clinical plans. The GO plans had slightly higher mean normal brain dose, but the difference was not statistically significant. The volume of normal brain receiving 6 Gy and higher was lower in the GO plans than in the clinical plans (Table 2 and Fig. 4). As an example, Fig. 5 displays the 2.1, 6.3, 10.5, and 21 Gy (prescription dose) isodose line for both the clinical plan and GO plan for a patient with eight lesions. Based on the treatment plans for these 10 patients, we found that the volume receiving intermediate to low dose (~6–15 Gy) is smaller in the GO plans than in the delivered clinical plans.

**Table 2:** Plan quality comparison between clinical plans and corresponding GO plans. Data show the range, average, standard deviation, and *P* value.

	Clinical plan	GO plan	*P* value
PTV			
Paddick‐GI	2.9–6.8 (4.4 ± 0.9)	2.8–6.0 (4.1 ± 0.9)	<0.0001
RTOG‐GI	3.3–9.1 (5.3 ± 1.3)	3.1–8.1 (4.9 ± 1.2)	<0.0001
CI	1.0–1.5 (1.2 ± 0.1)	1.0–1.4 (1.2 ± 0.1)	0.0056
Local V12Gy (cm^3^)	1.0–18.0 (5.5 ± 4.3)	0.9–18.0 (5.1 ± 4.2)	<0.0001
Normal brain			
Mean dose (cGy)	176.2–378.9 (265.1 ± 64.3)	191.5–381.2 (276.3 ± 66.6)	0.136
V4Gy (cm^3^)	103.8–475.8 (253.2 ± 112.2)	101.6–474.6 (249.5 ± 118.8)	0.732
V5Gy	68.8–277.7 (160.6 ± 75.9)	61.4–268.9 (148.5 ± 74.0)	0.419
V6Gy	49.1–208.2 (106.2 ± 51.8)	43.3–188.6 (96.2 ± 48.4)	0.01
V7Gy	37.2–136.4 (73.1 ± 32.6)	32.4–128.9 (67.0 ± 31.8)	0.004
V8Gy	29.1–93.4 (53.4 ± 21.9)	25.3–89.9 (49.5 ± 22.0)	0.002
V9Gy	23.2–68.6 (41.0 ± 16.1)	20.2–64.7 (37.9 ± 15.9)	0.002
V10Gy	18.8–52.3 (32.3 ± 12.4)	16.3–51.6 (29.7 ± 12.3)	0.002
V11Gy	15.3–41.9 (25.9 ± 9.7)	13.3–41.8 (23.7 ± 9.7)	0.002
V12Gy	12.5–34.2 (20.9 ± 7.7)	10.9–34.1 (19.2 ± 7.7)	0.002
V13Gy	10.0–28.0 (16.9 ± 6.1)	9.0–27.9 (15.6 ± 6.2)	0.002
V14Gy	8.1–22.9 (13.7 ± 4.8)	7.5–22.8 (12.7 ± 4.9)	0.001
V15Gy	6.5–18.5 (11.0 ± 3.8)	6.2–18.5 (10.3 ± 3.9)	0.002
V16Gy	5.2–14.9 (8.8 ± 3.0)	5.1–14.8 (8.2 ± 3.1)	0.003

## 4 DISCUSSION

Utilizing single isocenter VMAT planning to treat multiple intracranial metastases has been investigated in several publications.^1^, ^2^, ^3^, ^4^, ^5^, ^6^, ^7^ Thomas et al.^7^ found that single isocenter VMAT plans had equivalent conformity and dose gradient compared to Gamma Knife for 28 cases with 113 lesions. Other publications have questioned if single‐isocenter VMAT plans can provide equivalent plan quality. Published studies have found that the target coverage is reduced with increasing distance from the isocenter,^9^, ^11^ and any residual rotational setup error will reduce the coverage further.^10^ At our institution, we have found that due to dose modeling limitations by the treatment planning system, the AAA model does not provide accurate dose calculation for both the 2.5 mm and the 5 mm MLC on the TrueBeam STx and we chose to fit the model to the small MLC only and limit the treatment delivery to these leaves.^12^ For these reasons, there may be a rationale for using multiple isocenters, depending on the spatial distribution of the cranial metastases. The GO software provides fast grouping of lesions, optimal collimator angles for each arc, and the outcome is more standardized plan quality than is provided manually by a group of treatment planners. Since the release of the GO script in February 2019, it has been used for planning of more than 70 multi‐lesion SRS VMAT cases with three or more lesions at our institution.

Several prior studies present clustering solutions.^9^, ^15^ Morrison et al.^9^ manually assigned targets to one of the isocenters iteratively until the distance between the centroid of each target to the respective isocenter was less than 5 cm and overall distance was minimized. Yock et al.^15^ were the first group who applied a data clustering algorithm to solve lesion grouping and isocenter placement for multiple intracranial metastases by utilizing the k‐means clustering algorithm. In their study, SSED and target coverage metric were used as quantitative optimization objectives. However, they ran k‐means clustering from 1 isocenter up to m isocenters where m is the number of lesions and determined the optimal isocenter numbers by “finding the elbow” in the graphs of the objectives versus the isocenter number where diminishing returns became obvious. This may not be the most efficient way to find the balance between number of isocenters and plan quality. In our approach, we minimized the number of isocenters by starting the search from 1 isocenter. The number of isocenters will be increased only when the grouped lesions do not meet the geometric criteria. The default limits for X and Y can be adjusted so that in some cases, the number of isocenters can be reduced by increasing the limit for the distance check. This will result in a solution where some of the PTVs are outside the jaws for a part of the arc rotation, but the final treatment plans may still be clinically acceptable. Increasing the number of isocenters reduces the total SSED. However, our plan quality comparison indicates that more isocenters do not necessarily result in better plan qualities in terms of CI, GI, and normal brain dose. The relationship between the number of isocenters and plan quality is not linear and strongly depends on the lesion distribution. Ruggieri et al.^19^ demonstrated that single isocenter VMAT plans can have better plan qualities than multiple‐isocenter VMAT plans when geometry favors. Due to these facts, we chose to use a distance criteria for SRS VMAT planning and aimed to produce plans with fewer isocenters to reduce both planning and delivery time while providing similar or better plan quality.

Both the k‐means clustering and the exhaustive search algorithms use the minimum SSED to group the lesions, leading to the implicit assumption that SSED is similar for each group. This may lead to unfavorable clustering where adjacent lesions could be clustered in a different group. To solve this issue, we could use a different distance as variable and metric, for example a correlation distance^20^; or we could use another clustering algorithm like a density‐based algorithm^21^ or an expectation‐maximization algorithm.^22^ However, based on our experience with a few cases (data not shown), none of these methods can perfectly solve all possible distributions of lesions. Therefore, a hybrid method to provide multiple optimal cluster selections for the planners may be beneficial for very complex lesion distributions.

Finding the optimal collimator angle for each arc is critical to reduce dose to normal brain and critical organs resulting from the “island blocking problem” and larger than necessary jaw openings.^8^, ^14^ Kang, et al.^14^ proposed a method for collimator angle optimization for multi‐metastases VMAT planning by computing the overlapping sinogram between lesions. We chose instead to develop a method similar to Wu's^8^ in which the area of the MLC openings is calculated based on the projection of all lesions in the group, allowing us to minimize not only the unblocked area but also the area to cover lesions simultaneously. Minimizing the MLC opening necessary to cover lesions in this way would potentially reduce dose outside lesions and overlap between lesions. We do find a small but significant plan quality improvement for the cases where the GO plans used the same lesion grouping and arc geometry as the clinical plans but with the collimator angles optimized by the GO script.

In a few prior studies, the collimator optimization objectives have been used to also optimize couch angles.^8^, ^14^, ^15^, ^23^ In this study, we found that the objectives’ value in the collimator optimization was very similar for different couch angles and therefore decided it was not realistic to use the same algorithm for collimator optimization to also select best couch angles (data not shown). Furthermore, using only one simple objective may be not enough to optimize couch angle, and other objectives or optimization methods should be considered. Regardless, in 8 out of 10 test cases, 4 standard arc angles were used and produced slightly better plans than the clinical plans. This may indicate that for VMAT plans with multiple isocenters, optimal couch angles may not have a significant impact on the plan quality.

For cases with a large number of lesions, complex lesion distribution, and proximation between lesions and organs at risk, different human dosimetrists typically generate plans with very different lesion groupings, arc geometries, and collimator angles. The solution depends on the dosimetrists' experience level. It is not trivial to determine the optimal grouping of lesions. Some dosimetrists would create more isocenters and that would significantly increase both planning and delivery time but not necessarily create a better plan quality than a solution with fewer isocenters. Our goal was to automate the planning processes and significantly reduce the planning time while maintaining similar or better plan quality.

## 5 CONCLUSION

The GO script was implemented in the clinic in February 2019, and since then it has been used in the planning of more than 70 cases. Using the GO software to group PTVs, set isocenter, and optimize the collimator angles for all arcs resulted in similar or slightly improved plan quality as compared to the manually created clinical plans while significantly shortening planning time and providing more consistent plan quality among different planners.

## CONFLICT OF INTEREST

There is no conflict of interest related to this study.
